# Epidemiological, clinical, and laboratory factors associated with chronic kidney disease in Mexican HIV-infected patients

**DOI:** 10.1590/2175-8239-JBN-2018-0024

**Published:** 2018-07-10

**Authors:** Edgar Dehesa Lopez, Carlos Córdova-Cázarez, Rafael Valdez-Ortiz, Carlie Michelle Cardona-Landeros, María Fernanda Gutiérrez-Rico

**Affiliations:** 1 Hospital Civil de Culiacan, Centro de Investigación y Docencia en Ciencias de la Salud, Culiacan, Sinaloa, Mexico.; 2 Instituto Mexicano del Seguro Social, Culiacan, Sinaloa, Mexico.; 3 Hospital Civil de Culiacan, Culiacan, Sinaloa, Mexico.; 4 Hospital General de Mexico, Ciudad de Mexico, Mexico.

**Keywords:** Renal Insufficiency, Chronic, HIV, Renal Insufficiency, Insuficiência Renal Crônica, HIV, Insuficiência Renal

## Abstract

**Aim:**

To determine the prevalence of chronic kidney disease (CKD) and the epidemiological, clinical, and laboratory factors associated with CKD in Mexican HIV-infected patients.

**Methods:**

Cross-sectional study. We included 274 patients with HIV/AIDS. CKD was defined by the estimated glomerular filtration rate (eGFR < 60 mL/min/1.73 m^2^ assessed by CKD-EPI) and albuminuria criteria from KDIGO guidelines. Clinical, epidemiological, and laboratory characteristics were compared between patients with and without CKD. The factors associated with CKD were assessed by logistic regression analysis.

**Results:**

The mean age was 41±11 years, and 72.3% of the patients were men. The global prevalence of CKD was 11.7% (n = 32); 7.2% (n = 20) were defined by eGFR criterion; 7.6% (n = 21), by the albuminuria criterion; and 3.2% (n = 9), by both CKD criteria. The most frequently observed stages of CKD were KDIGO G3A1 stage with 4.7% (n = 13), KDIGO G1A2 stage with 3.6% (n = 10) and KDIGO G3A2 stage with 1.7% (n = 5). The factors associated with CKD were use of abacavir/lamivudine (OR 3.2; 95% CI 1.1-8.9; *p* = 0.03), a CD4 lymphocyte count < 400 cells/µL (OR 2.6; 95% 1.03-6.4, *p* = 0.04), age (OR 1.1; 95% CI 1.04-1.2, *p* = 0.001) and albuminuria (OR 19.98; 95% CI: 5.5-72.2; *p* < 0.001).

**Conclusions:**

CKD was a frequent complication in HIV-infected patients. These findings confirm the importance of screening and the early detection of CKD, as well as the importance of identifying and treating traditional and non-traditional risk factors associated with CKD.

## RESUMO

**Objetivo:**

Determinar a prevalência de doença renal crônica (DRC) e os fatores epidemiológicos, clínicos e laboratoriais associados à DRC em pacientes mexicanos infectados pelo HIV.

**Métodos:**

Estudo transversal. Incluímos 274 pacientes com HIV/AIDS. A DRC foi definida pela taxa de filtração glomerular estimada (TFGe < 60 mL/min/1,73 m^2^, avaliada pelo CKD-EPI) e pelos critérios de albuminúria das diretrizes do KDIGO. As características clínicas, epidemiológicas e laboratoriais foram comparadas entre pacientes com e sem DRC. Os fatores associados à DRC foram avaliados por análise de regressão logística.

**Resultados:**

A média da idade foi de 41 ± 11 anos e 72,3% dos pacientes eram homens. A prevalência global de DRC foi de 11,7% (n = 32); 7,2% (n = 20) foram definidos pelo critério TFGe; 7,6% (n = 21), pelo critério da albuminúria; e 3,2% (n = 9), pelos dois critérios para DRC. Os estágios mais frequentemente observados da DRC foram o estágio KDIGO G3A1 com 4,7% (n = 13); estágio KDIGO G1A2 com 3,6% (n = 10) e estágio KDIGO G3A2 com 1,7% (n = 5). Os fatores associados à DRC foram o uso de abacavir/lamivudina (OR 3,2; IC95% 1,1-8,9; *p* = 0,03), contagem de linfócitos CD4 < 400 células/µL (OR 2,6; 95% 1,03-6,4, *p* = 0,04), idade (OR 1,1; IC95% 1,04-1,2, *p* = 0,001) e albuminúria (OR 19,98; IC95%: 5,5-72,2; *p* < 0,001).

**Conclusões:**

A DRC foi uma complicação frequente em pacientes infectados pelo HIV. Esses achados confirmam a importância do rastreamento e da detecção precoce da DRC, bem como a importância de identificar e tratar os fatores de risco tradicionais e não tradicionais associados à DRC.

## INTRODUCTION

Significant advances in antiretroviral therapy have deceased the progression of disease and improved survival of HIV-infected patients. In fact, chronic kidney disease (CKD) has arisen as one of the leading noninfectious conditions affecting HIV-infected persons.^1^ The reported prevalence of CKD in patients infected with HIV in North America and Europe ranges from 4.7% to 9.7%, and higher rates have been reported when CKD is defined by either estimated glomerular filtration rate (eGFR) or proteinuria.^2^^-^ 4 HIV infection is a well-established risk factor for CKD and end-stage renal disease (ESRD).

The majority of CKD cases in HIV infection are due to HIV-associated nephropathy (HIV-AN). However, up to 50% of kidney diseases in HIV-infected persons result from a wide array of non-HIVAN pathology.^5^ These patients may develop multiple glomerular nephropathies (IgA nephropathy, lupus-like glomerulonephritis, focal and segmental glomerulosclerosis, membranoproliferative glomerulonephritis with cryoglobulinemia, and membranous glomerulopathy) and vascular (thrombotic microangiopathy), tubulointerstitial (tubular nephropathy by drugs, tubulointerstitial immunoallergic nephritis, and Fanconi syndrome), and obstructive nephropathies (nephropathy due to crystal deposition) related to the virus itself, the drugs administered or the coinfections.^6^ On the other hand, traditional risk factors for CKD are becoming increasingly prevalent in HIV-infected populations, including aging, diabetes *mellitus*, hypertension, cardiovascular disease, previous AKI and race/ethnicity.^7^ This has caused the risk factors for CKD in HIV-infected persons to be a combination of traditional and HIV-related factors, including low CD4 counts, high viral load, intravenous drug use, hepatitis C virus (HCV) coinfection, and use of specific antiretroviral drugs.^8^^-^ 12 Finally, as in the general population, albuminuria and decreased kidney function in HIV-positive individuals have been associated with worse outcomes such as a progression to AIDS and death.^13^^,^ 14

The aim of our study was to assess the prevalence of CKD and to determine epidemiological, clinical, and laboratory factors associated with CKD in Mexican HIV-infected patients.

## METHODS

A cross-sectional study was performed with patients evaluated from November 2015 to September 2016. The protocol was reviewed and approved by the Institutional Research and Ethics Committee. We included patients with HIV/AIDS from the Outpatient Center for AIDS and Sexual Transmission Infections Care (CAPASITS) in Sinaloa, Mexico.

Clinical (age, sex, smoking, drug use, medications, HCV coinfection, hepatitis B virus (HBV) coinfection, hypertension, and diabetes *mellitus* diagnosis), laboratory (serum creatinine, urea, hemoglobin, leukocytes, platelets, albuminuria, and estimated glomerular filtration rate) and HIV-infection variables (CD4 lymphocyte count, viral load, clinical stage of HIV-infection, antiretroviral medications) were collected and compared between patients with and without CKD.

The diagnosis of CKD was documented if the patient had two consecutive determinations, measured with a 3-month interval or more, of a GFR < 60 mL/min/1.73 m^2^ estimated with the equation CKD-EPI (GFR = 141 * min (Scr/κ,1) α * max ( Scr/κ, 1) -1.209 * 0.993Age * 1.018 [if female] * 1.159 [if black]) and/or albuminuria in the dipstick or 24-hour urine collection. The stage of CKD was determined by the eGFR rate (stage 1: eGFR > 90 mL/min/1.73 m^2^; stage 2: eGFR 60-89 mL/min/1.73 m^2^; stage 3a: eGFR 45-59 mL/min/1.73 m^2^; stage 3b: eGFR 30-44 mL/min/1.73 m^2^; stage 4: eGFR 15-29 mL/min/1.73 m^2^; stage 5: eGFR < 15 mL/min/1.73 m^2^) and albuminuria (A1: albuminuria < 30 mg/g; A2: albuminuria 30- 299 mg/g; A3: albuminuria > 300 mg/g), according to the diagnostic and classification criteria for CKD of the Kidney Disease Improving Global Outcomes (KDIGO) foundation.^15^^,^ 16 Staging of HIV infection was done according to clinical categories (category A1: asymptomatic infection, acute infection, persistent generalized lymphadenopathy; category B: symptomatic infection, not A or C; category C: defining conditions of AIDS) and immunological categories (category 1: lymphocyte count > 500 cells/µL; category 2: lymphocyte count of 200-499 cells/µL; category 3: lymphocyte count < 200 cells /µL) of the revised classification system of HIV infection of the Centers for Disease Control.^17^

Descriptive statistics with means and standard deviations were used to describe continuous variables; frequencies and proportions were used to describe categorical variables. Comparisons between groups were performed using Student's *t*-test and one-way ANOVA for continuous variables and the *χ* ^2^ test for categorical variables. The study of factors associated with CKD was performed by multivariate logistic regression. Variables with *p* < 0.05 in the univariate analysis and those that have been consistently associated with CKD in the medical literature were included in the multivariate analysis. A value of *p* < 0.05 was considered statistically significant.

## RESULTS

### 1. GENERAL POPULATION CHARACTERISTICS

We included 274 patients with an average age of 41 ± 11 years; of these patients, 72.3% (n = 198) were males. The most frequently observed comorbidities were smoking in 28.5% (n = 78), drug abuse in 14.6% (n = 40), hypertension in 7.7% (n = 21), and diabetes *mellitus* in 6.9% (n = 19) of the patients. The main viral coinfection in our population was HCV in 2.9% (n = 8) of the patients.

### 2. CLINICAL AND IMMUNOLOGICAL STAGES OF HIV-INFECTION

The most common clinical category was category B with 39.8% (n = 109), followed by category C with 31.4% (n = 86), and category A with 28.8% (n = 79) of the patients. The most common immunologic category was category 2 with 43.5% (n = 119), followed by category 3 with 40.1% (n = 110), and category 1 with 16.4% (n = 45) of the patients. Based on these categories, the most frequently observed clinical stages of HIV in our population were stage C3 (22.6%, n = 62), stage B2 (21.2%, n = 58), and stage A2 (15%, n = 41).

On the other hand, the initial viral load (pretreatment) was 89.220 + 244.945 copies/mL and the CD4 lymphocyte count was 369 + 276 cells/µL. Coinfection was observed in 8.3% (n = 23) of the HIV patients, of which 0.7% (n = 2) corresponded to coinfection with HBV; 2.9% (n = 8), to coinfection with HCV; and 4.7% (n = 13), to concomitant infection with *Treponema Pallidum.*

### 3. PREVALENCE OF CHRONIC KIDNEY DISEASE

The global prevalence of CKD in our HIV-infected population was 11.7% (n = 32); 7.2% (n = 20) were diagnosed by the eGFR criterion, 7.6% (n = 21) were diagnosed by the albuminuria criterion, and 3.2% (n = 9) by both CKD criteria. Of the patients with CKD, 34.3% (n = 11) were fulfilled with the eGFR < 60 mL/min criterion, 37.5% (n = 12) fulfilled the albuminuria criterion, and 28.1% (n = 9) with both CKD criteria.

The most frequent stages of CKD in our population were KDIGO G3A1 stage with 4.7% (n = 13), KDIGO G1A2 stage with 3.6% (n = 10), and KDIGO G3A2 stage with 1.7% (n = 5) of the cases (Table 1).

**Table 1:** Prevalence of CKD in Mexican HIV-infected patients

		Albuminuria KDIGO categories		
		A1	A2	A3
		n (%)	n (%)	n (%)
eGFR Categories	G1	2 (0.7%)	10 (3.6%)	
	G2			
	G3a	13 (4.7%)	4 (1.4%)	
	G3b		1 (0.3%)	
	G4			
	G5		2 (0.7%)	

CKD = Chronic kidney disease; eGFR = estimated glomerular filtration rate.

### 4. COMPARISON OF CLINICAL, LABORATORY, TREATMENT, AND HIV-INFECTION CHARACTERISTICS BETWEEN PATIENTS WITH AND WITHOUT CKD

When comparing the general characteristics between CKD patients vs non-CKD patients, we observed that patients with albuminuria-CKD criterion were younger than patients with eGFR-CKD criterion (35 *vs*. 51 years, *p* = 0.001), eGFR + albuminuria CKD criteria (35 *vs*. 49, *p* = 0.001), and non-CKD patients (35 vs 40, *p* = 0.001). No statistically significant difference was observed in the other general characteristics shown in Table 2.

**Table 2:** Comparison of clinical, laboratory and HIV-infection characteristics between patients with and without CKD

VARIABLES	No CKD		eGFR < 60 mL/min		Albuminuria (Alb)		eGFR < 60 mL/min + Alb		*p*
	n = 242	%	n = 11	%	n = 12	%	n = 9	%	
Age (years)	40 ± 11^a^		51 ± 10		35 ± 10^b,c,d^		49 ± 9^d^		0.001
Female	67	27.7%	3	27.3%	3	25.0%	3	33.3%	0.98
Male	175	72.3%	8	72.7%	9	75.0%	6	66.7%	
Smoking	70	28.9%	3	27.3%	2	16.7%	3	33.3%	0.81
Drug use	36	14.9%	1	9.1%	1	8.3%	2	22.2%	0.78
Hypertension	16	6.6%	2	18.2%	1	8.3%	2	22.2%	0.18
Diabetes *mellitus* 2	18	7.4%	0	0.0%	0	0.0%	1	11.1%	0.56
HIV viral load	88,937 ± 253,253		110,339 ± 187,174		29,086 ± 52,838		151,196 ± 239,386		0,71
CD4 count	371 ± 275		279 ± 167		407 ± 378		362 ± 294		0,70
Clinical HIV category									
A	71	29.3%	2	18.2%	6	50.0%	0	0,0%	0.26
B	94	38.8%	6	54.5%	4	33.3%	5	55.6%	
C	77	31.8%	3	27.3%	2	16.7%	4	44.4%	
Inmunological HIV category									
1	40	16.5%	2	18.2%	3	25,0%	0	0,0%	0.43
2	102	42.1%	4	36.4%	7	58.3%	6	66.7%	
3	100	41.3%	5	45.5%	2	16.7%	3	33.3%	
HBV Positive	1	0.4%	1	9.1%^a^	0	0.0%	0	0.0%	0.01
HCV Positive	7	2.9%	1	9.1%	0	0.0%	0	0.0%	0.55
VDRL Positive	10	4.1%	1	9.1%	1	8.3%	1	11.1%	0.61

HIV= human inmodeficiency virus; CKD = chronic kidney disease; eGFR = estimated glomerular filtration rate; HBV: hepatitis B virus; HCV: hepatitis C virus; VDRL: venereal disease research laboratory.

a) No CKD vs. eGFR < 60 ml/min;

b) albuminuria vs. No CKD;

c) albuminuria vs. eGFR< 60 ml/min;

d) albuminuria vs. eGFR< 60 ml/min + Albuminuria.

Comparing medical prescriptions between groups, we observed that the use of angiotensin II receptor antagonism (ARA) (11.1%, n = 1 *vs*. 0.8%, n = 2; *p* < 0.01) and antiretroviral treatment with saquinavir/ritonavir (22.2%, n = 2 *vs*. 3.3%, n = 8; *p* = 0.03) were more frequent in patients with CKD by eGFR and /or albuminuria criteria than in patients without CKD. No statistically significant difference was found for the remaining medications shown in Table 3.

**Table 3:** Comparison of medications used between patients with and without CKD

VARIABLES	No CKD		eGFR < 60mL/min		Albuminúria (Alb)		eGFR < 60mL/min + Alb		*p*
	n = 242	%	n = 11	%	n = 12	%	n = 9	%	
ACEI	12	5.0%	1	9.1%	0	0,0%	1	11.1%	0.64
ARA	2	0.8%	1	9.1%	1	8.3%	1	11.1%	< 0.01
Efavirenz + Tenofovir + Emtricitabine	84	34.7%	1	9.1%	3	25,0%	2	22.2%	0.26
Tenofovir + Emtricitabine	96	39.7%	5	45.5%	6	50,0%	4	44.4%	0.87
Atazanavir + Ritonavir	109	45.0%	7	63.6%	7	58.3%	1	11.1%	0.08
Abacavir/Lamivudine	50	20.7%	4	36.4%	3	25.0%	4	44.4%	0.23
Lopinavir/Ritonavir	20	8.3%	1	9.1%	0	0.0%	1	11.1%	0.75
Saquinavir + Ritonavir	8	3.3%	1	9.1%	1	8.3%	2	22.2%	0.03
Zidovudina + Lamivudine	3	1.2%	0	0.0%	0	0.0%	0	0.0%	0.94
Darunavir + Raltegravir + Ritonavir	5	2.1%	0	0.0%	0	0.0%	1	11.1%	0.27

CKD: Chronic kidney disease; eGFR: Estimated glomerular rate by CKD-EPI; ACEI: Angiotensin-converting enzyme inhibitor; ARA: Angiotensin II receptor antagonist.

### 5. FACTORS ASSOCIATED WITH CKD

In the multivariate logistic regression analysis, the factors associated with CKD defined by the eGFR and/or albuminuria in our HIV population were abacavir /lamivudine treatment, with an OR of 3.2 (95% CI of 1.1-8.9; p=0.03), and a CD4 lymphocyte count < 400 cells/µL, with an OR of 2.6 (95% CI of 1.03-6.4, *p* = 0.04). On the other hand, the factors associated with CKD defined only by the eGFR were age, with OR of 1.1 (95% of CI 1.04-1.2, *p* = 0.001); and albuminuria, with an OR of 19.98 (95% of CI: 5.5-72.2; *p* = 0.00). In our HIV population, we did not observe factors associated with CKD defined by albuminuria (Table 4).

**Table 4:** Factors asociated with CKD in Mexican HIV-infected patients

Variables	CKD by eGFR and/or albuminuria				CKD by eGFR < 60 mL/min				CKD by albuminuria			
	OR	CI 95%		*p*	OR	IC 95%		*p*	OR	CI 95%		*p*
		Lower	Upper			Lower	Upper			Lower	Upper	
Age (years)	1	0.9	1.1	0.13	1.1	1.04	1.2	0.001	1	0.95	1.03	0.8
Hypertension (yes/no)	0.5	0.02	11.4	0.68	0.3	0.004	29.8	0.6	0.6	0.02	23.4	0.8
Diabetes mellitus 2 (yes/no)	0.2	0.02	1.7	0.14	0.2	0.02	2.3	0.2	0.4	0.05	4.2	0.5
Saquinavir/ritonavir	3.3	0.8	13.3	0.09	3	0.5	17.4	0.2	3.5	0.8	16.3	0.1
Abacavir/lamivudine	3.2	1.1	8.9	0.03	2.2	0.5	9.8	0.3	2.7	0.8	9.2	0.1
Tenofovir/emtricitabine	2.2	0.9	5.6	0.09	1.5	0.4	5.9	0.6	2.2	0.7	6.6	0.2
ACEI or ARA	4.5	0.2	92.9	0.33	8.1	0.09	717.9	0.4	3.2	0.1	113.6	0.5
CD4 count (< 400 *vs*. > 400)	2.6	1.03	6.4	0.04	2.7	0.7	9.8	0.1	1.8	0.7	5.2	0.2
Albuminuria (yes/no)					19.98	5.5	72.2	< 0.01				

CKD = Chronic kidney disease, eGFR = Estimated glomerular rate by CKD-EPI, CI = Confidence intervale; OR = Odds ratio; ACEI = Angiotensin-converting enzyme inhibitor, ARA = Angiotensin II receptor antagonist.

## DISCUSSION

The global prevalence of CKD in our HIV population was 11.7% (n = 32); 7.2% (n = 20) of the patients were diagnosed by the eGFR criterion, and 7.6% (n = 21) of the patients were diagnosed by the albuminuria criterion. These findings are in line with previous reports by other researchers worldwide, ranging from 7.2% (USA) to 13.7% (China) applying the albuminuria criterion and from 3.5% (Europe) to 9.7% (USA) considering the eGFR criterion < 60 mL/min. On the other hand, the global prevalence of CKD (eGFR and /or albuminuria) in our population was less than that reported in Japan (15.5%) and USA (23.7%), although globally, this varies from 2 to 30%.^18^

Coinfection with HCV was 2.9% (n = 8), which was lower than previous reports (30%, with a range between 3-70%).^19^ On the other hand, HBV coinfection was 0.7% (n = 2) in our population, which is below the prevalence described by other authors. 20 ^,^ 21 Raboni *et al*. conducted a cross-sectional study in southern Brazil in which the authors documented hepatitis/HIV coinfection in 6.6% of HIV-infected patients. The HCV/HIV coinfection was confirmed in 4.0% and HBV/HIV coinfection in 1.3% of HIV-infected patients.^22^

Previous clinical studies have described multiple traditional risk factors (age, hypertension, diabetes, proteinuria, etc.) and non-traditional risk factors specific to HIV patients (elevated viral load, low CD4 cell count, coinfection with HBV and HCV, drugs, etc.) associated with the development of CKD.^7^^-^ 11

Age was a factor associated with CKD in our population. This finding is similar to those reported by other studies, being that the relative risk increase for CKD (defined as eGFR or albuminuria) for every 10 years of age is from 1.5 to 5.5. When age was analyzed as a dichotomous variable by other authors, patients > 50 years presented a relative risk of 2.0 for progression to ESRD or an eGFR < 15 mL/min compared to patients < 30 years.^16^

A CD4 lymphocyte count < 400 cells/µL was another factor associated with CKD (defined by GFR or albuminuria) in our population. This has been consistently demonstrated by other investigators, reporting a relative risk range for CKD (defined by GFR or albuminuria) from 1.1 to 1.25 for each decrease of 100 cells/µL or from 1.4 to 2.2 for patients with CD4 < 200 *vs*. > 201 cells/µL. On the other hand, the CD4 count has also been associated with the progression to ESRD or a GFR < 15 mL/min, with a relative risk of 1.7 per 100 cells/µL decrease and 1.4 to 2.7-fold increased risk for patients with CD4 < 200 *vs*. > 201 cells/µL.^16^ Another study documented a decreased CKD risk in association with CD4 cell restoration and plasma viral load suppression during treatment with HAART.^23^

In our HIV population, the presence of albuminuria was the main factor associated with CKD (defined by the eGFR criterion). The presence of albuminuria is frequent and is observed in up to a third of patients infected with HIV. Multiple observational studies in the general population and cohorts of HIV-infected patients demonstrate a strong and consistent association between the presence of albuminuria and clinical outcomes in these patients, such as progression to ESRD, cardiovascular disease, AIDS, and death.^14^^,^ 24 ^-^ 27

Treatment with abacavir/lamivudine was another factor associated with CKD in our population. Although abacavir and lamivudine have shown a low nephrotoxicity in some clinical studies, abacavir has been associated with tubular nephrotoxicity and acute tubulointerstitial nephritis and lamivudine with Fanconi syndrome and nephrogenic diabetes insipidus.^28^^,^ 29 Takeshi Nishijima *et al*. compared the deterioration of eGFR (a 25% decrease in baseline renal function) between HIV-infected patients treated with an antiretroviral regimen based on a tenofovir- *vs*. an abacavir-based regimen in a cohort of 503 Japanese patients. Although impairment of renal function was greater in patients under the tenofovir regimen (22.1%), impairment of renal function was also observed in patients under the abacavir regimen (13.5%).^30^ In the AIDS Clinical Trials Group Study 5202 (ACTG), the frequency of Fanconi syndrome, toxic nephropathy, proteinuria, and renal failure was similar between patients treated with tenofovir/emtricitabine and those treated with abacavir/lamivudine.^31^ These findings confirm that although abacavir has a lower nephrotoxicity profile, it is still a nephrotoxic antiretroviral drug.

Treatment with saquinavir/ritonavir (OR 3.3, 95% CI 0.8-13.3, *p* = 0.09) and tenofovir/emtricitabine (OR 2.2, 95% CI 0.9-5.6, *p* = 0.09) showed a statistical trend for CKD risk defined by eGFR and albuminuria. These findings have been confirmed by Mocroft *et al*., who studied the cumulative nephrotoxic effect of the major antiretroviral drugs used in a cohort of 23,005 HIV-infected patients in the United States, Europe, and Australia. The authors documented an increase in the risk of CKD (defined by eGFR < 60 mL/min) of 14, 20, and 11% for each year of exposure only for tenofovir, ritonavir/atazanavir and ritonavir/lopinavir, respectively. The increase in the incidence of CKD with chronic exposure to these antiretrovirals over the 6-year follow-up, indicates a cumulative nephrotoxic effect of tenofovir, ritonavir/atazinovir, and ritonavir/lopinavir.^32^ However, saquinavir has been shown to be a safe antiretroviral drug from the point of view of nephrotoxicity, so the risk of CKD of the saquinavir/ritonavir combination observed in our population is probably due to ritonavir. 33

Regarding the weaknesses of our work, the retrospective nature of the study stands out with all the limitations that this entails for obtaining clinical, laboratory, and antiretroviral treatment information. It was not possible to assess the effect of coinfection with HBV and HCV in the logistic regression model due to the small number of patients coinfected with these viruses in our population. It was also not possible to assess the effect of other nephrotoxic drugs frequently used by HIV-infected patients, such as trimetropim/sulfamethoxazole, acyclovir, amphotericin B, and NSAIDs.

## CONCLUSIONS

CKD was a frequent complication in the studied Mexican HIV-infected patients. The presence of albuminuria, age, CD4+ lymphocyte count, and abacavir/lamivudine treatment were the factors associated with CKD in our population. These findings confirm the importance of screening and early detection of CKD, as well as the identification and treatment of traditional and non-traditional risk factors associated with CKD. This could prevent or slow further decline in kidney function and improve outcomes in HIV-infected patients.
